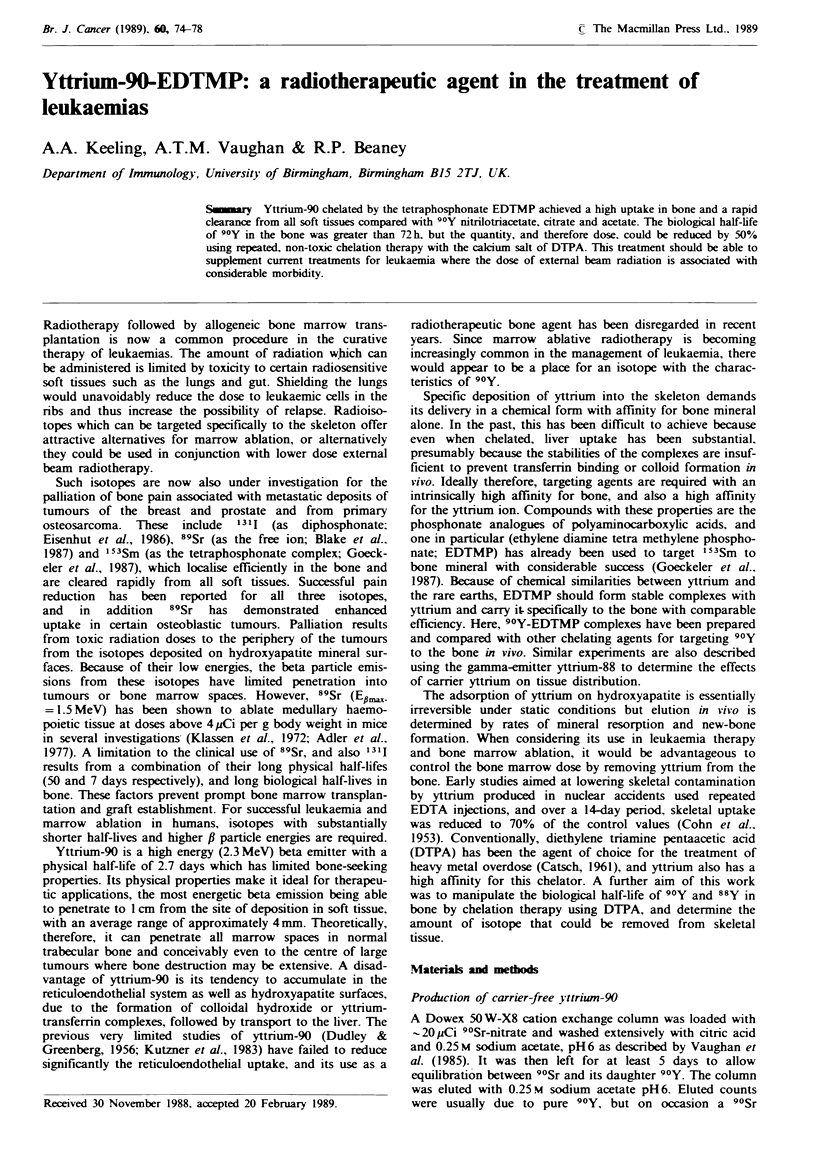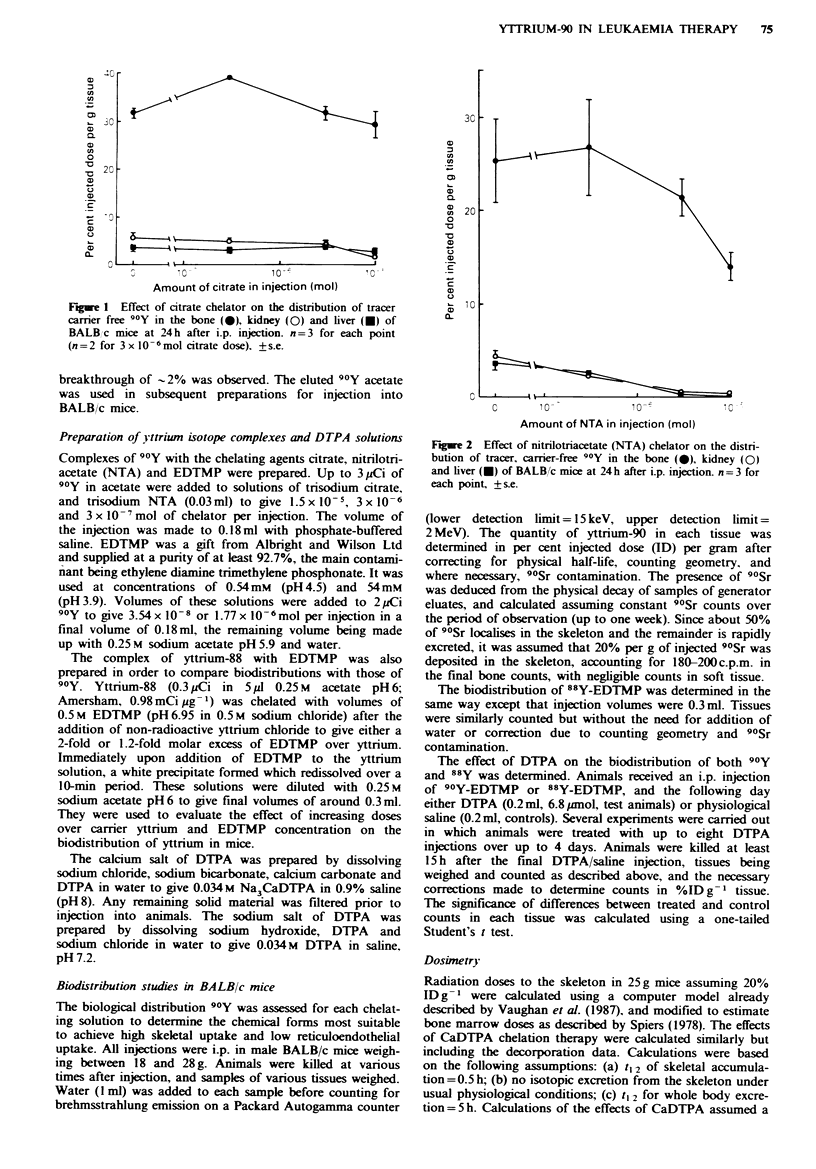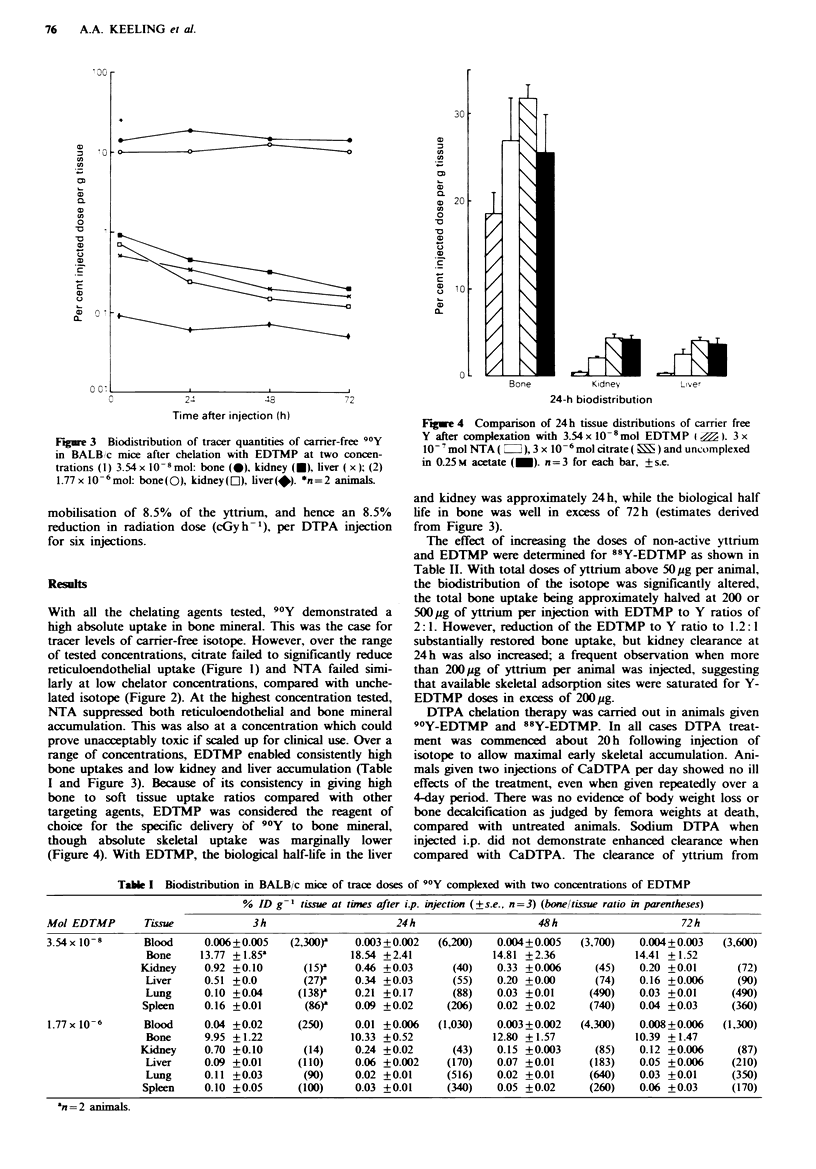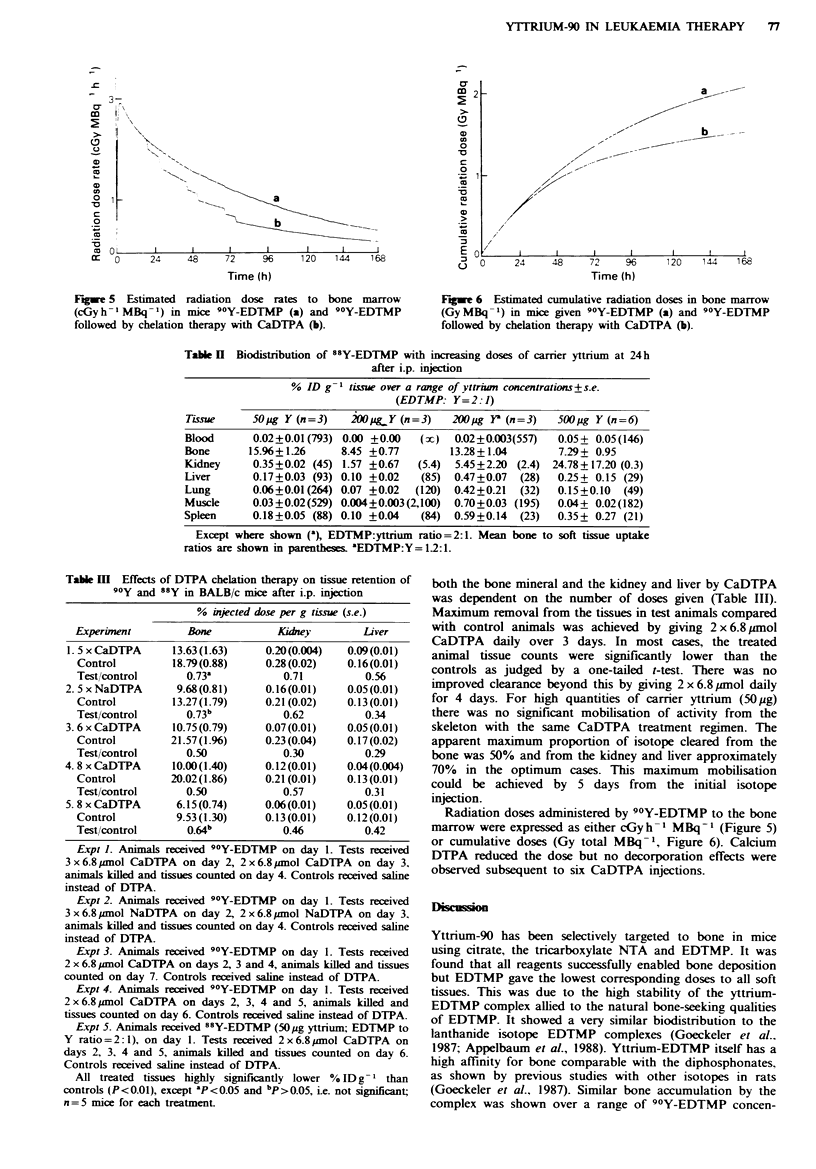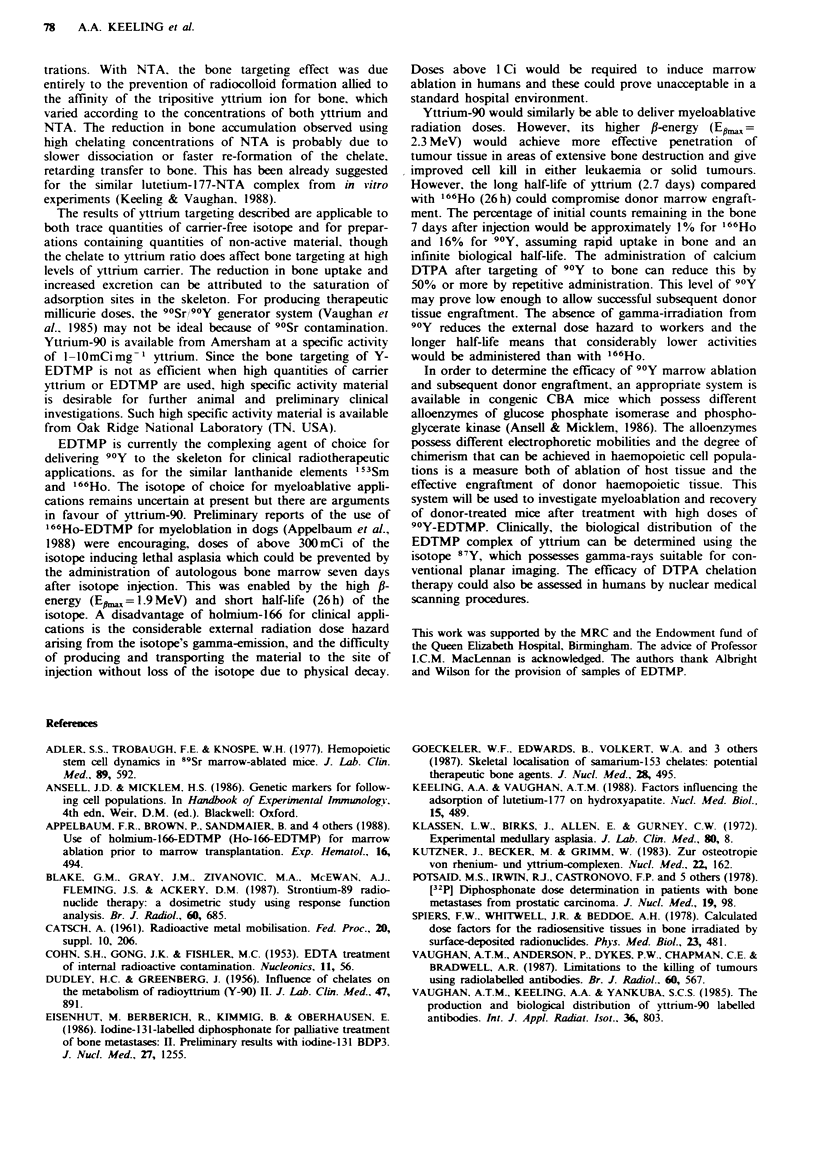# Yttrium-90-EDTMP: a radiotherapeutic agent in the treatment of leukaemias.

**DOI:** 10.1038/bjc.1989.223

**Published:** 1989-07

**Authors:** A. A. Keeling, A. T. Vaughan, R. P. Beaney

**Affiliations:** Department of Immunology, University of Birmingham, UK.

## Abstract

Yttrium-90 chelated by the tetraphosphonate EDTMP achieved a high uptake in bone and a rapid clearance from all soft tissues compared with 90Y nitrilotriacetate, citrate and acetate. The biological half-life of 90Y in the bone was greater than 72 h, but the quantity, and therefore dose, could be reduced by 50% using repeated, non-toxic chelation therapy with the calcium salt of DTPA. This treatment should be able to supplement current treatments for leukaemia where the dose of external beam radiation is associated with considerable morbidity.


Br. J. Cancer (1989), 60, 74–78 © The Macmillan Press Ltd., 1989

### Yttrium-90-EDTMP: a radiotherapeutic agent in the treatment of leukaemias

A.A. Keeling, A.T.M. Vaughan & R.P. Beaney

*Department of Immunology, University of Birmingham, Birmingham B15 2TJ, UK.*

**Summary** Yttrium-90 chelated by the tetraphosphonate EDTMP achieved a high uptake in bone and a rapid clearance from all soft tissues compared with $^{90}Y$ nitrilotriacetate, citrate and acetate. The biological half-life of $^{90}Y$ in the bone was greater than 72 h, but the quantity, and therefore dose, could be reduced by 50% using repeated, non-toxic chelation therapy with the calcium salt of DTPA. This treatment should be able to supplement current treatments for leukaemia where the dose of external beam radiation is associated with considerable morbidity.

Radiotherapy followed by allogeneic bone marrow transplantation is now a common procedure in the curative therapy of leukaemias. The amount of radiation which can be administered is limited by toxicity to certain radiosensitive soft tissues such as the lungs and gut. Shielding the lungs would unavoidably reduce the dose to leukaemic cells in the ribs and thus increase the possibility of relapse. Radioisotopes which can be targeted specifically to the skeleton offer attractive alternatives for marrow ablation, or alternatively they could be used in conjunction with lower dose external beam radiotherapy.

Such isotopes are now also under investigation for the palliation of bone pain associated with metastatic deposits of tumours of the breast and prostate and from primary osteosarcoma. These include $^{131}I$ (as diphosphonate; Eisenhut *et al.*, 1986), $^{89}Sr$ (as the free ion; Blake *et al.*, 1987) and $^{153}Sm$ (as the tetraphosphonate complex; Goeckeler *et al.*, 1987), which localise efficiently in the bone and are cleared rapidly from all soft tissues. Successful pain reduction has been reported for all three isotopes, and in addition $^{89}Sr$ has demonstrated enhanced uptake in certain osteoblastic tumours. Palliation results from toxic radiation doses to the periphery of the tumours from the isotopes deposited on hydroxyapatite mineral surfaces. Because of their low energies, the beta particle emissions from these isotopes have limited penetration into tumours or bone marrow spaces. However, $^{89}Sr$ ($E_{\beta max}$ = 1.5 MeV) has been shown to ablate medullary haemopoietic tissue at doses above 4 $\mu$Ci per g body weight in mice in several investigations (Klassen *et al.*, 1972; Adler *et al.*, 1977). A limitation to the clinical use of $^{89}Sr$, and also $^{131}I$ results from a combination of their long physical half-lifes (50 and 7 days respectively), and long biological half-lives in bone. These factors prevent prompt bone marrow transplantation and graft establishment. For successful leukaemia and marrow ablation in humans, isotopes with substantially shorter half-lives and higher $\beta$ particle energies are required.

Yttrium-90 is a high energy (2.3 MeV) beta emitter with a physical half-life of 2.7 days which has limited bone-seeking properties. Its physical properties make it ideal for therapeutic applications, the most energetic beta emission being able to penetrate to 1 cm from the site of deposition in soft tissue, with an average range of approximately 4 mm. Theoretically, therefore, it can penetrate all marrow spaces in normal trabecular bone and conceivably even to the centre of large tumours where bone destruction may be extensive. A disadvantage of yttrium-90 is its tendency to accumulate in the reticuloendothelial system as well as hydroxyapatite surfaces, due to the formation of colloidal hydroxide or yttrium-transferrin complexes, followed by transport to the liver. The previous very limited studies of yttrium-90 (Dudley & Greenberg, 1956; Kutzner *et al.*, 1983) have failed to reduce significantly the reticuloendothelial uptake, and its use as a radiotherapeutic bone agent has been disregarded in recent years. Since marrow ablative radiotherapy is becoming increasingly common in the management of leukaemia, there would appear to be a place for an isotope with the characteristics of $^{90}Y$.

Specific deposition of yttrium into the skeleton demands its delivery in a chemical form with affinity for bone mineral alone. In the past, this has been difficult to achieve because even when chelated, liver uptake has been substantial, presumably because the stabilities of the complexes are insufficient to prevent transferrin binding or colloid formation *in vivo*. Ideally therefore, targeting agents are required with an intrinsically high affinity for bone, and also a high affinity for the yttrium ion. Compounds with these properties are the phosphonate analogues of polyaminocarboxylic acids, and one in particular (ethylene diamine tetra methylene phosphonate; EDTMP) has already been used to target $^{153}Sm$ to bone mineral with considerable success (Goeckeler *et al.*, 1987). Because of chemical similarities between yttrium and the rare earths, EDTMP should form stable complexes with yttrium and carry it specifically to the bone with comparable efficiency. Here, $^{90}Y$-EDTMP complexes have been prepared and compared with other chelating agents for targeting $^{90}Y$ to the bone *in vivo*. Similar experiments are also described using the gamma-emitter yttrium-88 to determine the effects of carrier yttrium on tissue distribution.

The adsorption of yttrium on hydroxyapatite is essentially irreversible under static conditions but elution *in vivo* is determined by rates of mineral resorption and new-bone formation. When considering its use in leukaemia therapy and bone marrow ablation, it would be advantageous to control the bone marrow dose by removing yttrium from the bone. Early studies aimed at lowering skeletal contamination by yttrium produced in nuclear accidents used repeated EDTA injections, and over a 14-day period, skeletal uptake was reduced to 70% of the control values (Cohn *et al.*, 1953). Conventionally, diethylene triamine pentaacetic acid (DTPA) has been the agent of choice for the treatment of heavy metal overdose (Catsch, 1961), and yttrium also has a high affinity for this chelator. A further aim of this work was to manipulate the biological half-life of $^{90}Y$ and $^{88}Y$ in bone by chelation therapy using DTPA, and determine the amount of isotope that could be removed from skeletal tissue.

### Materials and methods

### *Production of carrier-free yttrium-90*

A Dowex 50 W-X8 cation exchange column was loaded with ~20 $\mu$Ci $^{90}Sr$-nitrate and washed extensively with citric acid and 0.25 M sodium acetate, pH 6 as described by Vaughan *et al.* (1985). It was then left for at least 5 days to allow equilibration between $^{90}Sr$ and its daughter $^{90}Y$. The column was eluted with 0.25 M sodium acetate pH 6. Eluted counts were usually due to pure $^{90}Y$, but on occasion a $^{90}Sr$

Received 30 November 1988, accepted 20 February 1989.

**Figure 1** Effect of citrate chelator on the distribution of tracer carrier free $^{90}Y$ in the bone (●), kidney (○) and liver (■) of BALB/c mice at 24 h after i.p. injection. $n = 3$ for each point ($n = 2$ for $3 \times 10^{-6}$ mol citrate dose). ±s.e.

breakthrough of ~2% was observed. The eluted $^{90}Y$ acetate was used in subsequent preparations for injection into BALB/c mice.

*Preparation of yttrium isotope complexes and DTPA solutions*

Complexes of $^{90}Y$ with the chelating agents citrate, nitrilotriacetate (NTA) and EDTMP were prepared. Up to 3 μCi of $^{90}Y$ in acetate were added to solutions of trisodium citrate, and trisodium NTA (0.03 ml) to give $1.5 \times 10^{-5}$, $3 \times 10^{-6}$ and $3 \times 10^{-7}$ mol of chelator per injection. The volume of the injection was made to 0.18 ml with phosphate-buffered saline. EDTMP was a gift from Albright and Wilson Ltd and supplied at a purity of at least 92.7%, the main contaminant being ethylene diamine trimethylene phosphonate. It was used at concentrations of 0.54 mM (pH 4.5) and 54 mM (pH 3.9). Volumes of these solutions were added to 2 μCi $^{90}Y$ to give $3.54 \times 10^{-8}$ or $1.77 \times 10^{-6}$ mol per injection in a final volume of 0.18 ml, the remaining volume being made up with 0.25 M sodium acetate pH 5.9 and water.

The complex of yttrium-88 with EDTMP was also prepared in order to compare biodistributions with those of $^{90}Y$. Yttrium-88 (0.3 μCi in 5 μl 0.25 M acetate pH 6; Amersham, 0.98 mCi $\mu g^{-1}$) was chelated with volumes of 0.5 M EDTMP (pH 6.95 in 0.5 M sodium chloride) after the addition of non-radioactive yttrium chloride to give either a 2-fold or 1.2-fold molar excess of EDTMP over yttrium. Immediately upon addition of EDTMP to the yttrium solution, a white precipitate formed which redissolved over a 10-min period. These solutions were diluted with 0.25 M sodium acetate pH 6 to give final volumes of around 0.3 ml. They were used to evaluate the effect of increasing doses over carrier yttrium and EDTMP concentration on the biodistribution of yttrium in mice.

The calcium salt of DTPA was prepared by dissolving sodium chloride, sodium bicarbonate, calcium carbonate and DTPA in water to give 0.034 M $Na_3CaDTPA$ in 0.9% saline (pH 8). Any remaining solid material was filtered prior to injection into animals. The sodium salt of DTPA was prepared by dissolving sodium hydroxide, DTPA and sodium chloride in water to give 0.034 M DTPA in saline, pH 7.2.

*Biodistribution studies in BALB/c mice*

The biological distribution $^{90}Y$ was assessed for each chelating solution to determine the chemical forms most suitable to achieve high skeletal uptake and low reticuloendothelial uptake. All injections were i.p. in male BALB/c mice weighing between 18 and 28 g. Animals were killed at various times after injection, and samples of various tissues weighed. Water (1 ml) was added to each sample before counting for brehmsstrahlung emission on a Packard Autogamma counter

**Figure 2** Effect of nitrilotriacetate (NTA) chelator on the distribution of tracer, carrier-free $^{90}Y$ in the bone (●), kidney (○) and liver (■) of BALB/c mice at 24 h after i.p. injection. $n = 3$ for each point, ±s.e.

(lower detection limit = 15 keV, upper detection limit = 2 MeV). The quantity of yttrium-90 in each tissue was determined in per cent injected dose (ID) per gram after correcting for physical half-life, counting geometry, and where necessary, $^{90}Sr$ contamination. The presence of $^{90}Sr$ was deduced from the physical decay of samples of generator eluates, and calculated assuming constant $^{90}Sr$ counts over the period of observation (up to one week). Since about 50% of $^{90}Sr$ localises in the skeleton and the remainder is rapidly excreted, it was assumed that 20% per g of injected $^{90}Sr$ was deposited in the skeleton, accounting for 180–200 c.p.m. in the final bone counts, with negligible counts in soft tissue.

The biodistribution of $^{88}Y$-EDTMP was determined in the same way except that injection volumes were 0.3 ml. Tissues were similarly counted but without the need for addition of water or correction due to counting geometry and $^{90}Sr$ contamination.

The effect of DTPA on the biodistribution of both $^{90}Y$ and $^{88}Y$ was determined. Animals received an i.p. injection of $^{90}Y$-EDTMP or $^{88}Y$-EDTMP, and the following day either DTPA (0.2 ml, 6.8 μmol, test animals) or physiological saline (0.2 ml, controls). Several experiments were carried out in which animals were treated with up to eight DTPA injections over up to 4 days. Animals were killed at least 15 h after the final DTPA/saline injection, tissues being weighed and counted as described above, and the necessary corrections made to determine counts in $\%ID\,g^{-1}$ tissue. The significance of differences between treated and control counts in each tissue was calculated using a one-tailed Student's $t$ test.

*Dosimetry*

Radiation doses to the skeleton in 25 g mice assuming 20% $ID\,g^{-1}$ were calculated using a computer model already described by Vaughan *et al.* (1987), and modified to estimate bone marrow doses as described by Spiers (1978). The effects of CaDTPA chelation therapy were calculated similarly but including the decorporation data. Calculations were based on the following assumptions: (a) $t_{1/2}$ of skeletal accumulation = 0.5 h; (b) no isotopic excretion from the skeleton under usual physiological conditions; (c) $t_{1/2}$ for whole body excretion = 5 h. Calculations of the effects of CaDTPA assumed a

**Figure 3** Biodistribution of tracer quantities of carrier-free $^{90}Y$ in BALB/c mice after chelation with EDTMP at two concentrations (1) $3.54 \times 10^{-8}$ mol: bone (●), kidney (■), liver (×); (2) $1.77 \times 10^{-6}$ mol: bone (○), kidney (□), liver (◆). *$n = 2$ animals.

**Figure 4** Comparison of 24 h tissue distributions of carrier free Y after complexation with $3.54 \times 10^{-8}$ mol EDTMP ( ), $3 \times 10^{-7}$ mol NTA ( ), $3 \times 10^{-6}$ mol citrate ( ) and uncomplexed in 0.25 M acetate ( ). $n = 3$ for each bar, ±s.e.

mobilisation of 8.5% of the yttrium, and hence an 8.5% reduction in radiation dose (cGy $h^{-1}$), per DTPA injection for six injections.

### Results

With all the chelating agents tested, $^{90}Y$ demonstrated a high absolute uptake in bone mineral. This was the case for tracer levels of carrier-free isotope. However, over the range of tested concentrations, citrate failed to significantly reduce reticuloendothelial uptake (Figure 1) and NTA failed similarly at low chelator concentrations, compared with unchelated isotope (Figure 2). At the highest concentration tested, NTA suppressed both reticuloendothelial and bone mineral accumulation. This was also at a concentration which could prove unacceptably toxic if scaled up for clinical use. Over a range of concentrations, EDTMP enabled consistently high bone uptakes and low kidney and liver accumulation (Table I and Figure 3). Because of its consistency in giving high bone to soft tissue uptake ratios compared with other targeting agents, EDTMP was considered the reagent of choice for the specific delivery of $^{90}Y$ to bone mineral, though absolute skeletal uptake was marginally lower (Figure 4). With EDTMP, the biological half-life in the liver and kidney was approximately 24 h, while the biological half life in bone was well in excess of 72 h (estimates derived from Figure 3).

The effect of increasing the doses of non-active yttrium and EDTMP were determined for $^{88}Y$-EDTMP as shown in Table II. With total doses of yttrium above 50 μg per animal, the biodistribution of the isotope was significantly altered, the total bone uptake being approximately halved at 200 or 500 μg of yttrium per injection with EDTMP to Y ratios of 2:1. However, reduction of the EDTMP to Y ratio to 1.2:1 substantially restored bone uptake, but kidney clearance at 24 h was also increased; a frequent observation when more than 200 μg of yttrium per animal was injected, suggesting that available skeletal adsorption sites were saturated for Y-EDTMP doses in excess of 200 μg.

DTPA chelation therapy was carried out in animals given $^{90}Y$-EDTMP and $^{88}Y$-EDTMP. In all cases DTPA treatment was commenced about 20 h following injection of isotope to allow maximal early skeletal accumulation. Animals given two injections of CaDTPA per day showed no ill effects of the treatment, even when given repeatedly over a 4-day period. There was no evidence of body weight loss or bone decalcification as judged by femora weights at death, compared with untreated animals. Sodium DTPA when injected i.p. did not demonstrate enhanced clearance when compared with CaDTPA. The clearance of yttrium from

**Table I** Biodistribution in BALB/c mice of trace doses of $^{90}Y$ complexed with two concentrations of EDTMP

| | | *% ID $g^{-1}$ tissue at times after i.p. injection (±s.e., n = 3) (bone/tissue ratio in parentheses)* | | | | | | | |
|---|---|---|---|---|---|---|---|---|---|
| *Mol EDTMP* | *Tissue* | *3 h* | | *24 h* | | *48 h* | | *72 h* | |
| $3.54 \times 10^{-8}$ | Blood | 0.006 ± 0.005 | (2,300)[a] | 0.003 ± 0.002 | (6,200) | 0.004 ± 0.005 | (3,700) | 0.004 ± 0.003 | (3,600) |
| | Bone | 13.77 ± 1.85[a] | | 18.54 ± 2.41 | | 14.81 ± 2.36 | | 14.41 ± 1.52 | |
| | Kidney | 0.92 ± 0.10 | (15)[a] | 0.46 ± 0.03 | (40) | 0.33 ± 0.006 | (45) | 0.20 ± 0.01 | (72) |
| | Liver | 0.51 ± 0.0 | (27)[a] | 0.34 ± 0.03 | (55) | 0.20 ± 0.00 | (74) | 0.16 ± 0.006 | (90) |
| | Lung | 0.10 ± 0.04 | (138)[a] | 0.21 ± 0.17 | (88) | 0.03 ± 0.01 | (490) | 0.03 ± 0.01 | (490) |
| | Spleen | 0.16 ± 0.01 | (86)[a] | 0.09 ± 0.02 | (206) | 0.02 ± 0.02 | (740) | 0.04 ± 0.03 | (360) |
| $1.77 \times 10^{-6}$ | Blood | 0.04 ± 0.02 | (250) | 0.01 ± 0.006 | (1,030) | 0.003 ± 0.002 | (4,300) | 0.008 ± 0.006 | (1,300) |
| | Bone | 9.95 ± 1.22 | | 10.33 ± 0.52 | | 12.80 ± 1.57 | | 10.39 ± 1.47 | |
| | Kidney | 0.70 ± 0.10 | (14) | 0.24 ± 0.02 | (43) | 0.15 ± 0.003 | (85) | 0.12 ± 0.006 | (87) |
| | Liver | 0.09 ± 0.01 | (110) | 0.06 ± 0.002 | (170) | 0.07 ± 0.01 | (183) | 0.05 ± 0.006 | (210) |
| | Lung | 0.11 ± 0.03 | (90) | 0.02 ± 0.01 | (516) | 0.02 ± 0.01 | (640) | 0.03 ± 0.01 | (350) |
| | Spleen | 0.10 ± 0.05 | (100) | 0.03 ± 0.01 | (340) | 0.05 ± 0.02 | (260) | 0.06 ± 0.03 | (170) |

[a] $n = 2$ animals.

**Figure 5** Estimated radiation dose rates to bone marrow (cGy h$^{-1}$ MBq$^{-1}$) in mice $^{90}$Y-EDTMP (**a**) and $^{90}$Y-EDTMP followed by chelation therapy with CaDTPA (**b**).

**Figure 6** Estimated cumulative radiation doses in bone marrow (Gy MBq$^{-1}$) in mice given $^{90}$Y-EDTMP (**a**) and $^{90}$Y-EDTMP followed by chelation therapy with CaDTPA (**b**).

**Table II** Biodistribution of $^{88}$Y-EDTMP with increasing doses of carrier yttrium at 24 h after i.p. injection

| | *% ID g$^{-1}$ tissue over a range of yttrium concentrations ± s.e. (EDTMP: Y = 2:1)* | | | |
|---|---|---|---|---|
| *Tissue* | *50 µg Y (n = 3)* | *200 µg Y (n = 3)* | *200 µg Y$^{a}$ (n = 3)* | *500 µg Y (n = 6)* |
| Blood | 0.02 ± 0.01 (793) | 0.00 ± 0.00 (∞) | 0.02 ± 0.003 (557) | 0.05 ± 0.05 (146) |
| Bone | 15.96 ± 1.26 | 8.45 ± 0.77 | 13.28 ± 1.04 | 7.29 ± 0.95 |
| Kidney | 0.35 ± 0.02 (45) | 1.57 ± 0.67 (5.4) | 5.45 ± 2.20 (2.4) | 24.78 ± 17.20 (0.3) |
| Liver | 0.17 ± 0.03 (93) | 0.10 ± 0.02 (85) | 0.47 ± 0.07 (28) | 0.25 ± 0.15 (29) |
| Lung | 0.06 ± 0.01 (264) | 0.07 ± 0.02 (120) | 0.42 ± 0.21 (32) | 0.15 ± 0.10 (49) |
| Muscle | 0.03 ± 0.02 (529) | 0.004 ± 0.003 (2,100) | 0.70 ± 0.03 (195) | 0.04 ± 0.02 (182) |
| Spleen | 0.18 ± 0.05 (88) | 0.10 ± 0.04 (84) | 0.59 ± 0.14 (23) | 0.35 ± 0.27 (21) |

Except where shown ($^{a}$), EDTMP:yttrium ratio = 2:1. Mean bone to soft tissue uptake ratios are shown in parentheses. $^{a}$EDTMP:Y = 1.2:1.

**Table III** Effects of DTPA chelation therapy on tissue retention of $^{90}$Y and $^{88}$Y in BALB/c mice after i.p. injection

| | *% injected dose per g tissue (s.e.)* | | |
|---|---|---|---|
| *Experiment* | *Bone* | *Kidney* | *Liver* |
| 1. 5 × CaDTPA | 13.63 (1.63) | 0.20 (0.004) | 0.09 (0.01) |
| Control | 18.79 (0.88) | 0.28 (0.02) | 0.16 (0.01) |
| Test/control | 0.73$^{a}$ | 0.71 | 0.56 |
| 2. 5 × NaDTPA | 9.68 (0.81) | 0.16 (0.01) | 0.05 (0.01) |
| Control | 13.27 (1.79) | 0.21 (0.02) | 0.13 (0.01) |
| Test/control | 0.73$^{b}$ | 0.62 | 0.34 |
| 3. 6 × CaDTPA | 10.75 (0.79) | 0.07 (0.01) | 0.05 (0.01) |
| Control | 21.57 (1.96) | 0.23 (0.04) | 0.17 (0.02) |
| Test/control | 0.50 | 0.30 | 0.29 |
| 4. 8 × CaDTPA | 10.00 (1.40) | 0.12 (0.01) | 0.04 (0.004) |
| Control | 20.02 (1.86) | 0.21 (0.01) | 0.13 (0.01) |
| Test/control | 0.50 | 0.57 | 0.31 |
| 5. 8 × CaDTPA | 6.15 (0.74) | 0.06 (0.01) | 0.05 (0.01) |
| Control | 9.53 (1.30) | 0.13 (0.01) | 0.12 (0.01) |
| Test/control | 0.64$^{b}$ | 0.46 | 0.42 |

*Expt 1.* Animals received $^{90}$Y-EDTMP on day 1. Tests received 3 × 6.8 µmol CaDTPA on day 2, 2 × 6.8 µmol CaDTPA on day 3, animals killed and tissues counted on day 4. Controls received saline instead of DTPA.

*Expt 2.* Animals received $^{90}$Y-EDTMP on day 1. Tests received 3 × 6.8 µmol NaDTPA on day 2, 2 × 6.8 µmol NaDTPA on day 3, animals killed and tissues counted on day 4. Controls received saline instead of DTPA.

*Expt 3.* Animals received $^{90}$Y-EDTMP on day 1. Tests received 2 × 6.8 µmol CaDTPA on days 2, 3 and 4, animals killed and tissues counted on day 7. Controls received saline instead of DTPA.

*Expt 4.* Animals received $^{90}$Y-EDTMP on day 1. Tests received 2 × 6.8 µmol CaDTPA on days 2, 3, 4 and 5, animals killed and tissues counted on day 6. Controls received saline instead of DTPA.

*Expt 5.* Animals received $^{88}$Y-EDTMP (50 µg yttrium; EDTMP to Y ratio = 2:1), on day 1. Tests received 2 × 6.8 µmol CaDTPA on days 2, 3, 4 and 5, animals killed and tissues counted on day 6. Controls received saline instead of DTPA.

All treated tissues highly significantly lower % ID g$^{-1}$ than controls ($P < 0.01$), except $^{a}P < 0.05$ and $^{b}P > 0.05$, i.e. not significant; $n = 5$ mice for each treatment.

both the bone mineral and the kidney and liver by CaDTPA was dependent on the number of doses given (Table III). Maximum removal from the tissues in test animals compared with control animals was achieved by giving 2 × 6.8 µmol CaDTPA daily over 3 days. In most cases, the treated animal tissue counts were significantly lower than the controls as judged by a one-tailed *t*-test. There was no improved clearance beyond this by giving 2 × 6.8 µmol daily for 4 days. For high quantities of carrier yttrium (50 µg) there was no significant mobilisation of activity from the skeleton with the same CaDTPA treatment regimen. The apparent maximum proportion of isotope cleared from the bone was 50% and from the kidney and liver approximately 70% in the optimum cases. This maximum mobilisation could be achieved by 5 days from the initial isotope injection.

Radiation doses administered by $^{90}$Y-EDTMP to the bone marrow were expressed as either cGy h$^{-1}$ MBq$^{-1}$ (Figure 5) or cumulative doses (Gy total MBq$^{-1}$, Figure 6). Calcium DTPA reduced the dose but no decorporation effects were observed subsequent to six CaDTPA injections.

### Discussion

Yttrium-90 has been selectively targeted to bone in mice using citrate, the tricarboxylate NTA and EDTMP. It was found that all reagents successfully enabled bone deposition but EDTMP gave the lowest corresponding doses to all soft tissues. This was due to the high stability of the yttrium-EDTMP complex allied to the natural bone-seeking qualities of EDTMP. It showed a very similar biodistribution to the lanthanide isotope EDTMP complexes (Goeckeler *et al.*, 1987; Appelbaum *et al.*, 1988). Yttrium-EDTMP itself has a high affinity for bone comparable with the diphosphonates, as shown by previous studies with other isotopes in rats (Goeckeler *et al.*, 1987). Similar bone accumulation by the complex was shown over a range of $^{90}$Y-EDTMP concentrations. With NTA, the bone targeting effect was due entirely to the prevention of radiocolloid formation allied to the affinity of the tripositive yttrium ion for bone, which varied according to the concentrations of both yttrium and NTA. The reduction in bone accumulation observed using high chelating concentrations of NTA is probably due to slower dissociation or faster re-formation of the chelate, retarding transfer to bone. This has been already suggested for the similar lutetium-177-NTA complex from *in vitro* experiments (Keeling & Vaughan, 1988).

The results of yttrium targeting described are applicable to both trace quantities of carrier-free isotope and for preparations containing quantities of non-active material, though the chelate to yttrium ratio does affect bone targeting at high levels of yttrium carrier. The reduction in bone uptake and increased excretion can be attributed to the saturation of adsorption sites in the skeleton. For producing therapeutic millicurie doses, the $^{90}Sr/^{90}Y$ generator system (Vaughan *et al.*, 1985) may not be ideal because of $^{90}Sr$ contamination. Yttrium-90 is available from Amersham at a specific activity of 1–10mCi mg$^{-1}$ yttrium. Since the bone targeting of Y-EDTMP is not as efficient when high quantities of carrier yttrium or EDTMP are used, high specific activity material is desirable for further animal and preliminary clinical investigations. Such high specific activity material is available from Oak Ridge National Laboratory (TN, USA).

EDTMP is currently the complexing agent of choice for delivering $^{90}Y$ to the skeleton for clinical radiotherapeutic applications, as for the similar lanthanide elements $^{153}Sm$ and $^{166}Ho$. The isotope of choice for myeloablative applications remains uncertain at present but there are arguments in favour of yttrium-90. Preliminary reports of the use of $^{166}$Ho-EDTMP for myeloblation in dogs (Appelbaum *et al.*, 1988) were encouraging, doses of above 300mCi of the isotope inducing lethal asplasia which could be prevented by the administration of autologous bone marrow seven days after isotope injection. This was enabled by the high $\beta$-energy ($E_{\beta max}$ = 1.9MeV) and short half-life (26h) of the isotope. A disadvantage of holmium-166 for clinical applications is the considerable external radiation dose hazard arising from the isotope's gamma-emission, and the difficulty of producing and transporting the material to the site of injection without loss of the isotope due to physical decay. Doses above 1Ci would be required to induce marrow ablation in humans and these could prove unacceptable in a standard hospital environment.

Yttrium-90 would similarly be able to deliver myeloablative radiation doses. However, its higher $\beta$-energy ($E_{\beta max}$ = 2.3MeV) would achieve more effective penetration of tumour tissue in areas of extensive bone destruction and give improved cell kill in either leukaemia or solid tumours. However, the long half-life of yttrium (2.7 days) compared with $^{166}Ho$ (26h) could compromise donor marrow engraftment. The percentage of initial counts remaining in the bone 7 days after injection would be approximately 1% for $^{166}Ho$ and 16% for $^{90}Y$, assuming rapid uptake in bone and an infinite biological half-life. The administration of calcium DTPA after targeting of $^{90}Y$ to bone can reduce this by 50% or more by repetitive administration. This level of $^{90}Y$ may prove low enough to allow successful subsequent donor tissue engraftment. The absence of gamma-irradiation from $^{90}Y$ reduces the external dose hazard to workers and the longer half-life means that considerably lower activities would be administered than with $^{166}Ho$.

In order to determine the efficacy of $^{90}Y$ marrow ablation and subsequent donor engraftment, an appropriate system is available in congenic CBA mice which possess different alloenzymes of glucose phosphate isomerase and phosphoglycerate kinase (Ansell & Micklem, 1986). The alloenzymes possess different electrophoretic mobilities and the degree of chimerism that can be achieved in haemopoietic cell populations is a measure both of ablation of host tissue and the effective engraftment of donor haemopoietic tissue. This system will be used to investigate myeloablation and recovery of donor-treated mice after treatment with high doses of $^{90}$Y-EDTMP. Clinically, the biological distribution of the EDTMP complex of yttrium can be determined using the isotope $^{87}Y$, which possesses gamma-rays suitable for conventional planar imaging. The efficacy of DTPA chelation therapy could also be assessed in humans by nuclear medical scanning procedures.

This work was supported by the MRC and the Endowment fund of the Queen Elizabeth Hospital, Birmingham. The advice of Professor I.C.M. MacLennan is acknowledged. The authors thank Albright and Wilson for the provision of samples of EDTMP.

**References**

ADLER, S.S., TROBAUGH, F.E. & KNOSPE, W.H. (1977). Hemopoietic stem cell dynamics in $^{89}Sr$ marrow-ablated mice. *J. Lab. Clin. Med.*, **89**, 592.

ANSELL, J.D. & MICKLEM, H.S. (1986). Genetic markers for following cell populations. In *Handbook of Experimental Immunology*, 4th edn, Weir, D.M. (ed.). Blackwell: Oxford.

APPELBAUM, F.R., BROWN, P., SANDMAIER, B. and 4 others (1988). Use of holmium-166-EDTMP (Ho-166-EDTMP) for marrow ablation prior to marrow transplantation. *Exp. Hematol.*, **16**, 494.

BLAKE, G.M., GRAY, J.M., ZIVANOVIC, M.A., McEWAN, A.J., FLEMING, J.S. & ACKERY, D.M. (1987). Strontium-89 radionuclide therapy: a dosimetric study using response function analysis. *Br. J. Radiol.*, **60**, 685.

CATSCH, A. (1961). Radioactive metal mobilisation. *Fed. Proc.*, **20**, suppl. 10, 206.

COHN, S.H., GONG, J.K. & FISHLER, M.C. (1953). EDTA treatment of internal radioactive contamination. *Nucleonics*, **11**, 56.

DUDLEY, H.C. & GREENBERG, J. (1956). Influence of chelates on the metabolism of radioyttrium (Y-90) II. *J. Lab. Clin. Med.*, **47**, 891.

EISENHUT, M. BERBERICH, R., KIMMIG, B. & OBERHAUSEN, E. (1986). Iodine-131-labelled diphosphonate for palliative treatment of bone metastases: II. Preliminary results with iodine-131 BDP3. *J. Nucl. Med.*, **27**, 1255.

GOECKELER, W.F., EDWARDS, B., VOLKERT, W.A. and 3 others (1987). Skeletal localisation of samarium-153 chelates: potential therapeutic bone agents. *J. Nucl. Med.*, **28**, 495.

KEELING, A.A. & VAUGHAN, A.T.M. (1988). Factors influencing the adsorption of lutetium-177 on hydroxyapatite. *Nucl. Med. Biol.*, **15**, 489.

KLASSEN, L.W., BIRKS, J., ALLEN, E. & GURNEY, C.W. (1972). Experimental medullary asplasia. *J. Lab. Clin. Med.*, **80**, 8.

KUTZNER, J., BECKER, M. & GRIMM, W. (1983). Zur osteotropie von rhenium- und yttrium-complexen. *Nucl. Med.*, **22**, 162.

POTSAID, M.S., IRWIN, R.J., CASTRONOVO, F.P. and 5 others (1978). [$^{32}P$] Diphosphonate dose determination in patients with bone metastases from prostatic carcinoma. *J. Nucl. Med.*, **19**, 98.

SPIERS, F.W., WHITWELL, J.R. & BEDDOE, A.H. (1978). Calculated dose factors for the radiosensitive tissues in bone irradiated by surface-deposited radionuclides. *Phys. Med. Biol.*, **23**, 481.

VAUGHAN, A.T.M., ANDERSON, P., DYKES, P.W., CHAPMAN, C.E. & BRADWELL, A.R. (1987). Limitations to the killing of tumours using radiolabelled antibodies. *Br. J. Radiol.*, **60**, 567.

VAUGHAN, A.T.M., KEELING, A.A. & YANKUBA, S.C.S. (1985). The production and biological distribution of yttrium-90 labelled antibodies. *Int. J. Appl. Radiat. Isot.*, **36**, 803.